# Etymologia: *Cochliomyia hominivorax*

**DOI:** 10.3201/eid2502.ET2502

**Published:** 2019-02

**Authors:** Ronnie Henry

**Keywords:** Cochliomyia hominivorax, New World screwworm fly, screwworm, larvae, insect, parasite, wounds, infestations, Charles Coquerel

## *Cochliomyia hominivorax* [kokʺle-o-miʹyǝ]

From the Greek *kochlias* (“snail with a spiral shell”) + *myia* (“fly”) and the Latin *hominis* (“man”) + *vorax* (“consuming”), *Cochliomyia hominivorax*, or the New World screwworm fly (formerly *Callitroga* [Greek *kallos*, “beautiful,” + *trogein*, “to gnaw”] *americana*) (Figure), was first described by French entomologist Charles Coquerel in 1858. *C. hominivorax* larvae enter wounds and feed on living tissue, and if untreated, infestations can be fatal. *C. hominivorax* was eliminated in the United States in 1982 and in much of Central America in the 1990s, although outbreaks associated with reimportations in infected humans and animals continue to occur.

**Figure:**
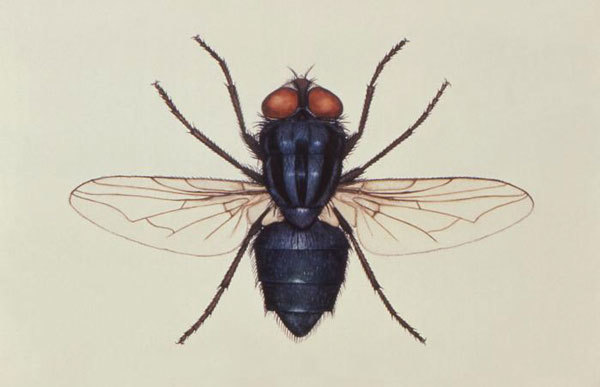
Depicts a dorsal view of the “Primary screwworm” fly, *Cochliomyia hominivorax*, a member of the family Calliphoridae. Image: Public Health Image Library.
